# Supplementary material for: Cancer-Associated Fibroblasts Modulate Transcriptional Signatures Involved in Proliferation, Differentiation and Metastasis in Head and Neck Squamous Cell Carcinoma
Source: Cancers (Basel). 2021 Jul 4;13(13):3361. doi: 10.3390/cancers13133361 (PMC8269044; doi:10.3390/cancers13133361)
Supplement: Supplementary file 1 [file cancers-13-03361-s001.zip › SUPPLEMENTARY/supplementary.pdf]

Supplementary Metatrail

Cancer-Associated Fibroblasts Modulate Transcriptional Signatures Involved in Proliferation, Differentiation and Metastasis in Head and Neck Squamous Cell Carcinoma

Emilia Wiechec, Mustafa Magan, Natasa Matic, Anna Ansell-Schultz, Matti Kankainen, Outi Monni, Ann-Charlotte Johansson and Karin Roberg

Table S1. Characteristics of the eight HNSCC cell lines.

Cell line	Gender	Localization	Clinical stage[a]
LK0824	M	Tongue	T2N1M0
LK0858	F	Tongue	T3N0M0
LK0902	F	Tongue	T1N0M0
LK0917	M	Gingiva	T4N1M1
LK0923	F	Larynx	T1N0M0
LK0942	M	Larynx	T4N0M0
LK0949	M	Tongue	T2N0M0
LK1108	F	Hypopharynx	T2N0M0

[a] TNM classification of primary tumors according to the International Union against Cancer (UICC, 2002).

Table S2. Clinical characteristics of patients.

Characteristics	Non responder N (%) (total=16)	Responder N (%) (total=16)
Sex		
Man	10 (62.5)	6 (37.5)
Woman	6 (37.5)	10 (62.5)
Age (years)		
Mean value	67	67.7
Range	37-86	55-81
Primary tumor site		
Gingiva	3 (18.8)	3 (18.8)
Larynx	4 (25)	3 (18.8)
Tongue	6 (37.5)	5 (31.3)
Trigonum retromolare	1 (6.3)	1 (6.3)
Buccal mucosa	2 (12.5)	1 (6.3)
Hypopharynx	0 (0)	1 (6.3)
Palate mucosa	0 (0)	1 (6.3)
Floor of mouth	0 (0)	1 (6.3)
Primary tumor (T)		
T2	6 (37.5)	8 (50)
T3	6 (37.5)	4 (25)
T4	4 (25)	3 (18.8)
Undetermined	0 (0)	1 (6.3)
Regional lymph nodes (N)		
N0	10 (62.5)	11 (68.8)
N1	2 (12.5)	0 (0)
N2	3 (18.8)	4 (25)
Undetermined	1 (6.3)	1 (6.3)

Distant metastasis (M)		
M0	16 (100)	15 (93.8)
Undetermined	0 (0)	1(6.3)
Differentiation grade		
High	5 (31.3)	2-3 (12.5-18.8)
Moderate	6-8 (37.5-50)	10-11 (62.5-68.8)
Low	3-5 (18.8-31.3)	3 (18.8)

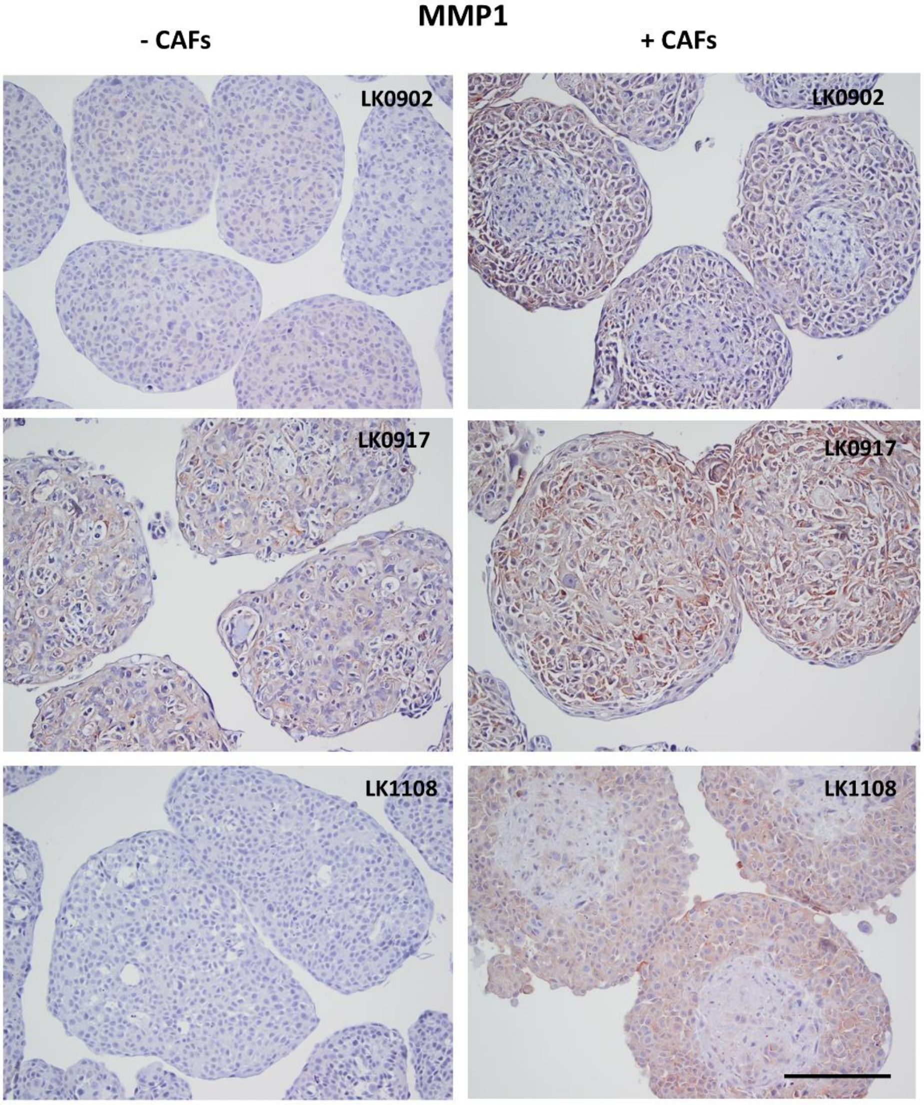

Figure S1. MMP1 expression in HNSCC cells grown in 3D ± CAFs. Immunohisto-chemical staining of HNSCC tumor spheroids ± CAFs with MMP1. Scale bar = 200 µm.

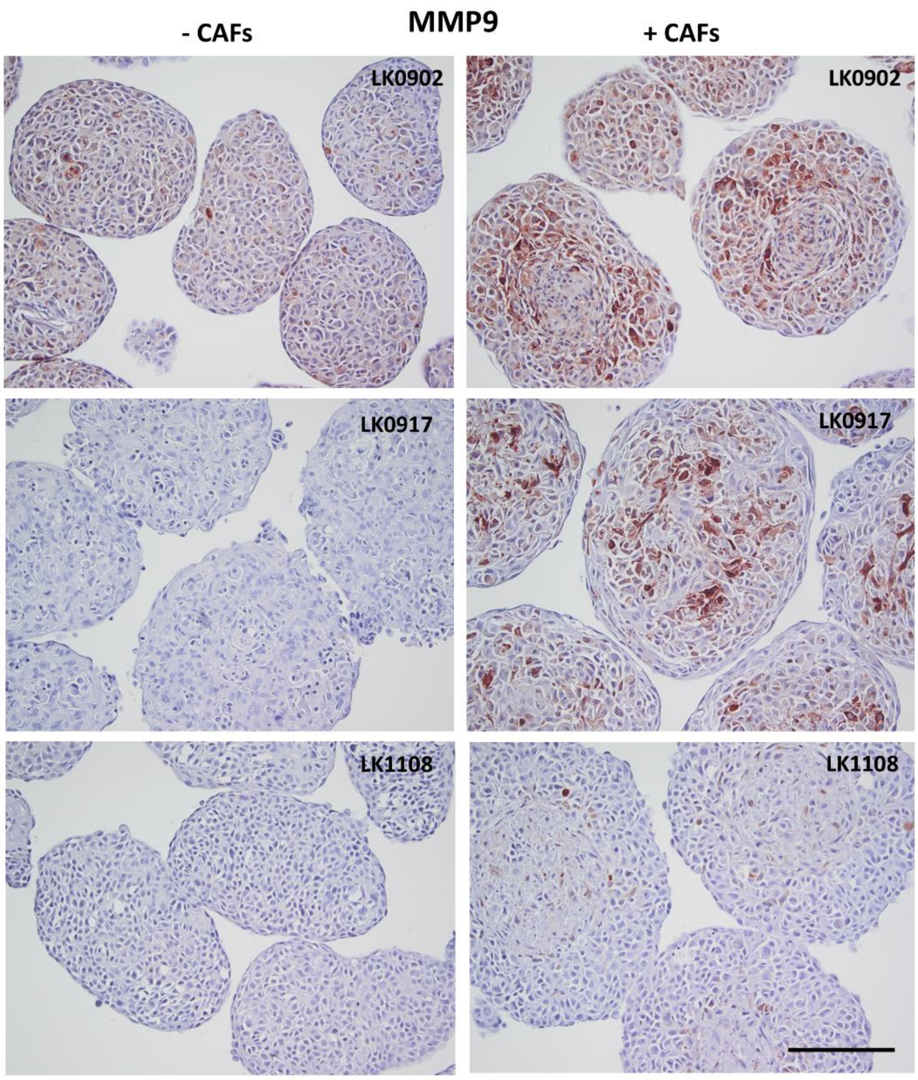

Figure S2. MMP9 expression in HNSCC cells grown in 3D ± CAFs. Immunohisto-chemical staining of HNSCC tumor spheroids ± CAFs with MMP9. Scale bar = 200 µm.

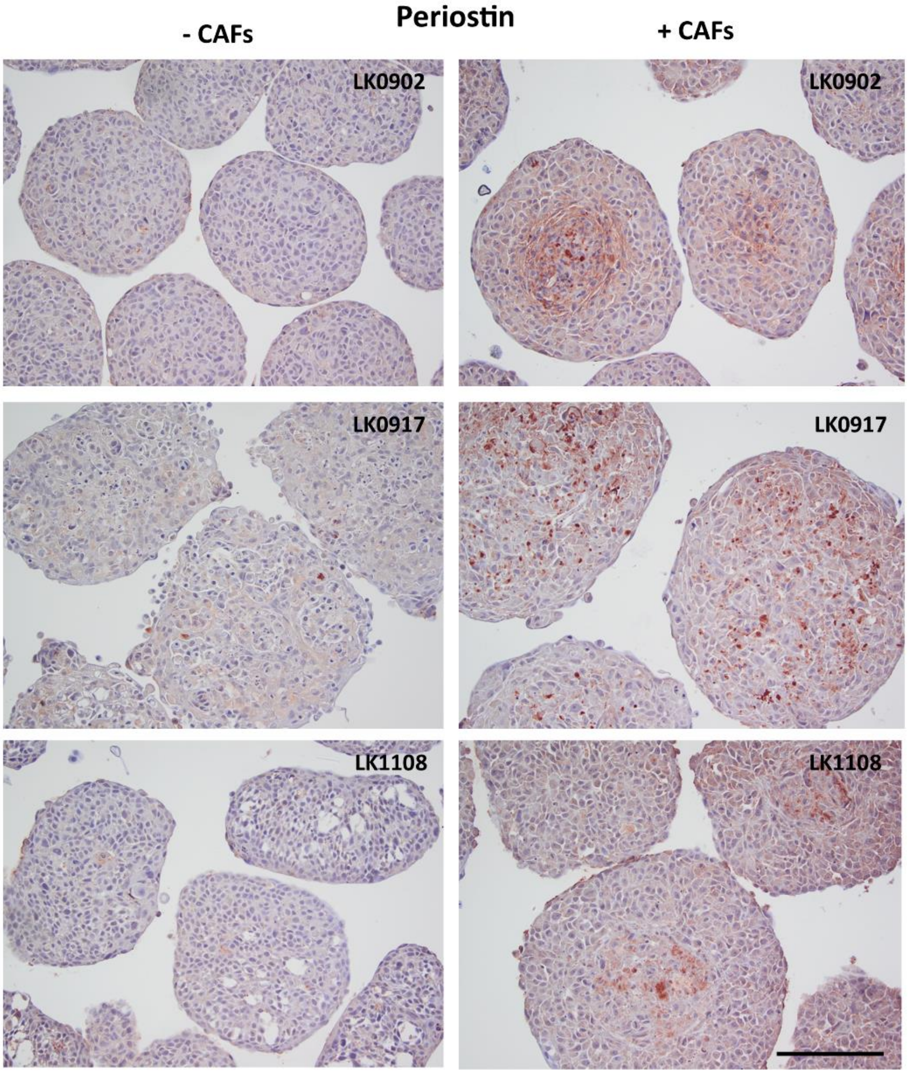

Figure S3. Periostin expression in HNSCC cells grown in 3D ± CAFs. Immunohistochemical staining of HNSCC tumor spheroids ± CAFs with periostin. Scale bar = 200 µm.

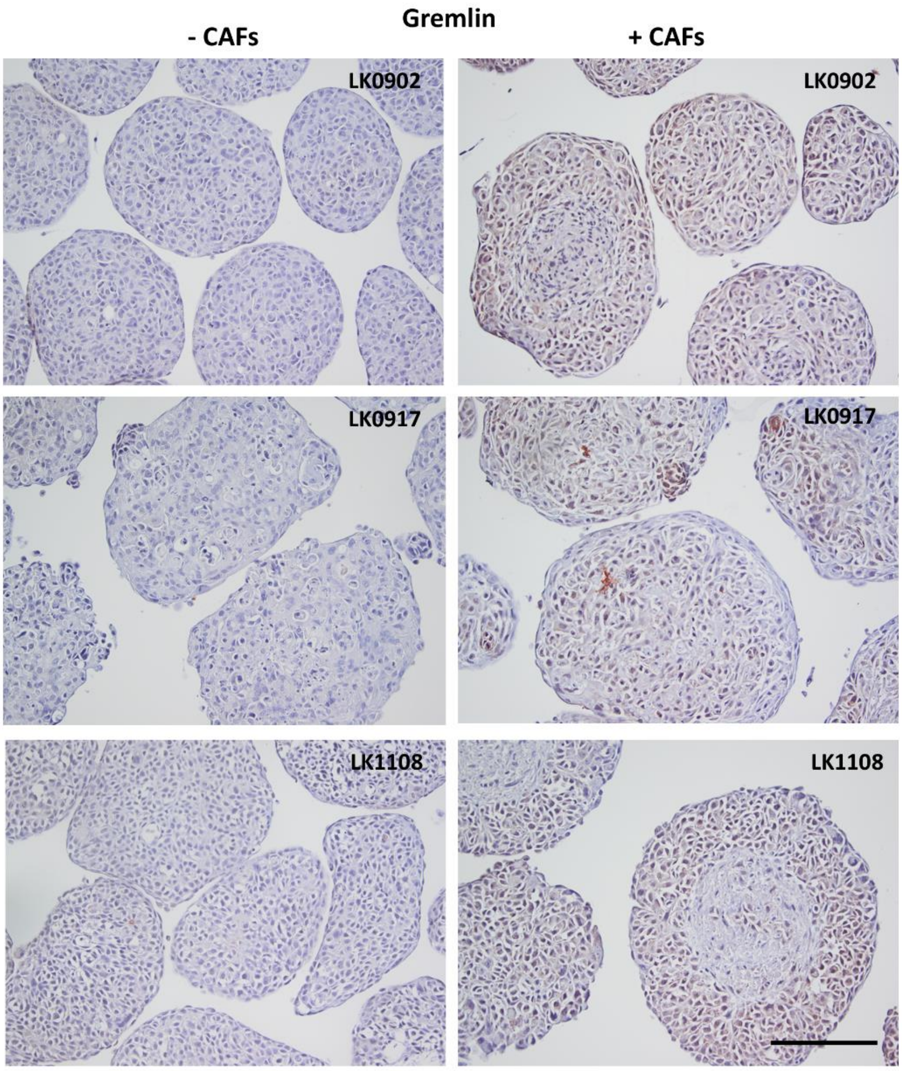

Figure S4. Gremlin expression in HNSCC cells grown in 3D ± CAFs. Immunohistochemical staining of HNSCC tumor spheroids ± CAFs with gremlin. Scale bar = 200 µm.

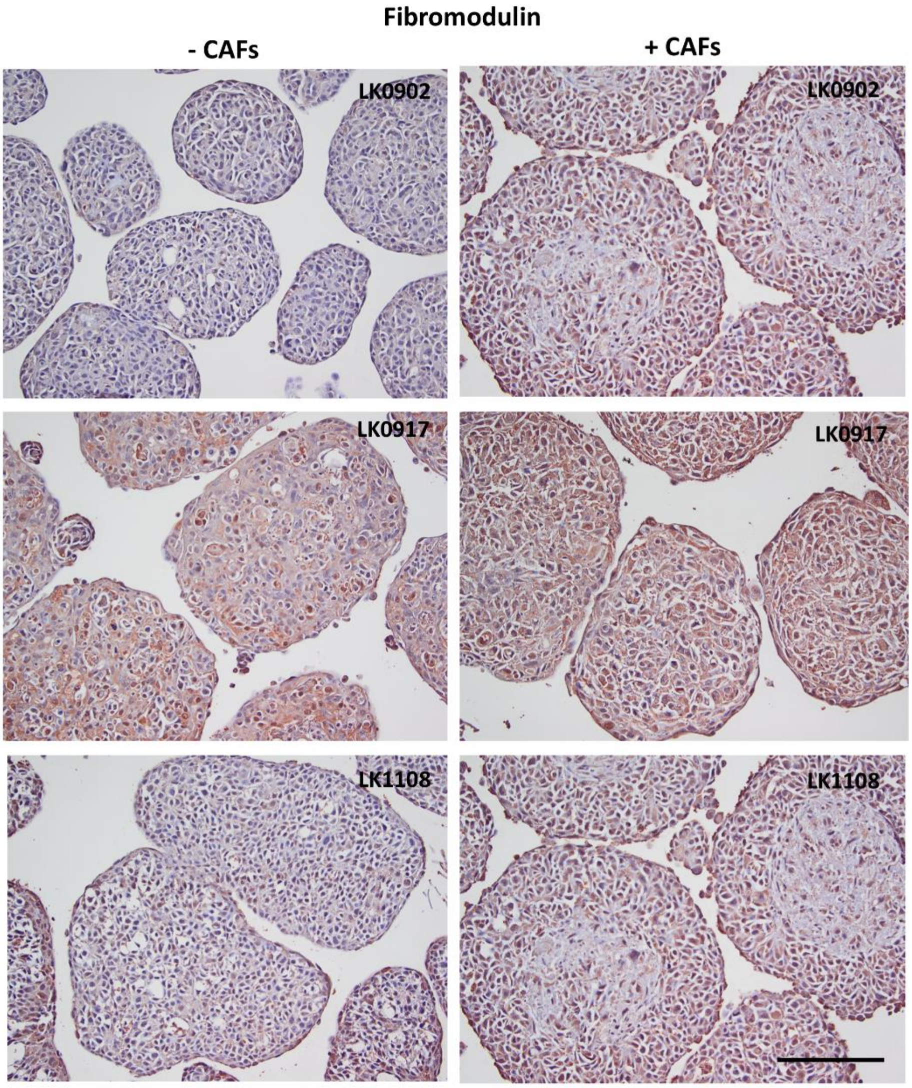

Figure S5. Fibromodulin expression in HNSCC cells grown in 3D ± CAFs. Immunohistochemical staining of HNSCC tumor spheroids ± CAFs with fibromodulin. Scale bar = 200 µm.

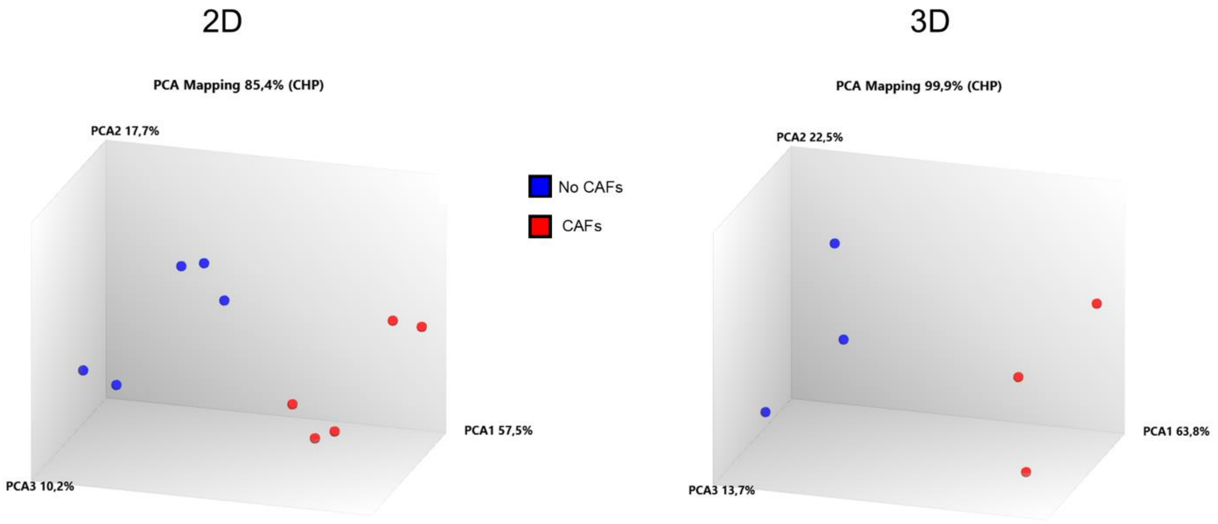

Figure S6: PCA plot from microarray analysis data of CAF vs NCAF in 2D and 3D model.

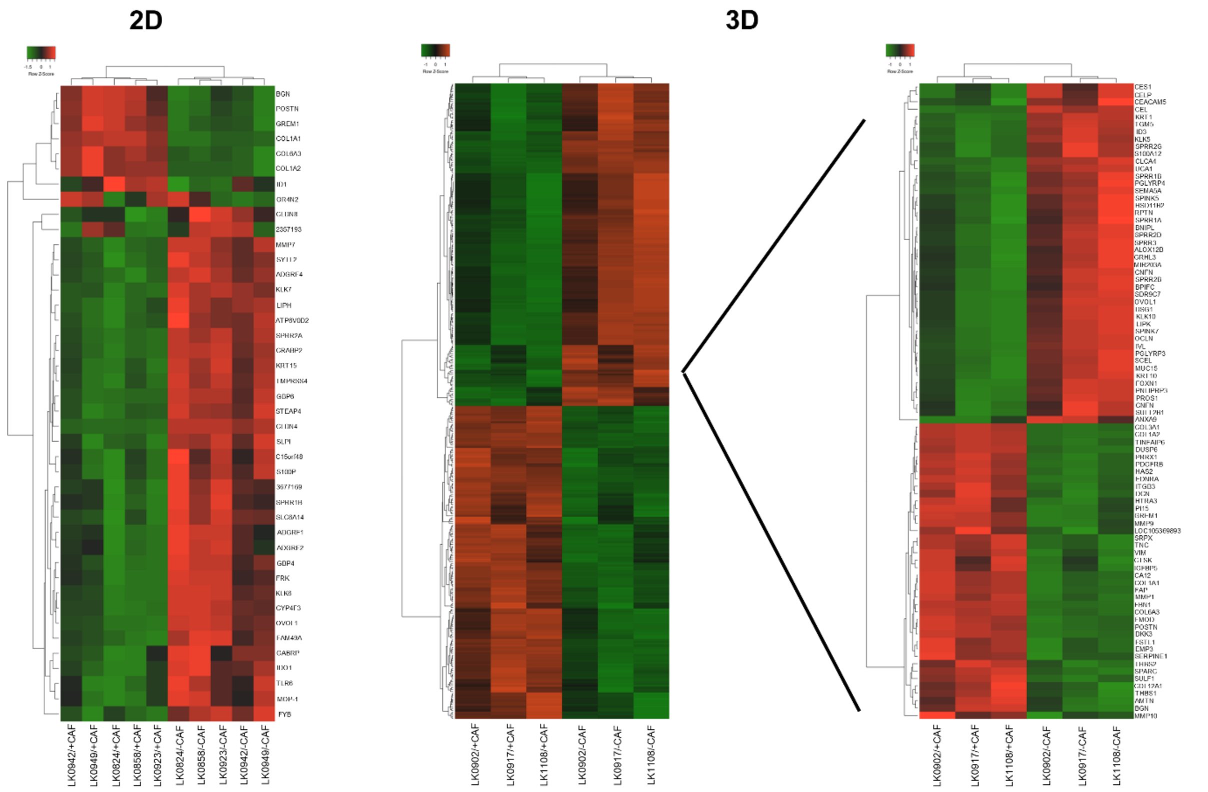

Figure S7: Representative heatmap of microarray expression data from HNSCC cells grown with and without CAF in 2D and 3D model. The color scale represents the raw z score ranging from red (high expression level) to green (low expression level).
